# Antibodies against Severe Fever with Thrombocytopenia Syndrome Virus in Healthy Persons, China, 2013

**DOI:** 10.3201/eid2008.131796

**Published:** 2014-08

**Authors:** Lei Zhang, Jimin Sun, Jie Yan, Huakun Lv, Chengliang Chai, Yi Sun, Bin Shao, Jianmin Jiang, Zhiping Chen, Jeroen Kortekaas, Yanjun Zhang

**Affiliations:** Zhejiang Provincial Center for Disease Control and Prevention, Hangzhou, China (L. Zhang, J. Sun, H. Lv, C. Chai, Y. Sun, B. Shao, J. Jiang, Z. Chen, Y. Zhang);; Central Veterinary Institute, part of Wageningen University and Research Centre, Lelystad, the Netherlands (J. Kortekaas);; Zhejiang University School of Medicine, Hangzhou (J. Yan)

**Keywords:** severe fever with thrombocytopenia syndrome virus, SFTSV, viruses, Zhejiang Province, China, seroprevalence, healthy persons, viruses

## Abstract

In June 2013, a subclinical infection with severe fever with thrombocytopenia syndrome virus (SFTSV) was detected in Zhejiang Province, China, prompting seroprevalence studies in 6 districts within the province. Of 986 healthy persons tested, 71 had IgG antibodies against SFTSV. This finding suggests that most natural infections with SFTSV are mild or subclinical.

Severe fever with thrombocytopenia syndrome (SFTS), a serious emerging infectious disease, was first reported in rural areas of central China in 2009 (*1*). The characteristic signs and symptoms of SFTS include fever, thrombocytopenia, and leukocytopenia, and the disease has a case-fatality rate of up to 30%. SFTS is caused by infection with SFTS virus (SFTSV; family *Bunyaviridae*, genus *Phlebovirus*). The virus was first isolated in 2010 from patients with SFTS (*1*); since then, additional cases have been reported from many areas of China (*2*,*3*). After the occurrence of SFTS cases in Zhejiang Province, China, in 2013, enhanced surveillance for the disease was implemented (*4*). We report on the first human case of SFTS in a rural area of Pujiang district in Zhejiang Province and on an apparently associated mild or subclinical case of SFTSV infection in a family member of the patient. In addition, to determine if other mild or subclinical infections had occurred, we conducted seroprevalence studies in the patient’s village and 5 other Zhejiang Province districts. The study was approved by the Ethics Committee of the Zhejiang Provincial Center for Disease Control and Prevention.

## The Study

**Figure 1:**
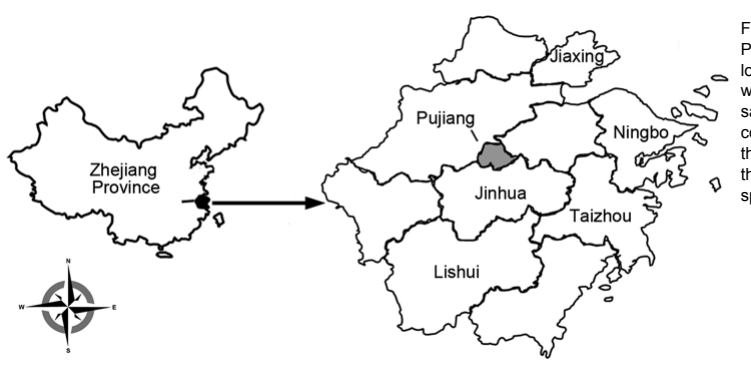
Location of Zhejiang Province in China (left) and the location of selected districts (right) within the province where serum samples of healthy persons were collected and tested in 2013 for the presence of severe fever with thrombocytopenia syndrome virus–specific IgG and IgM.

A human case of SFTS was identified in a rural area of the Pujiang district (Figure 1). The patient, a 60-year-old local male subsistence farmer, sought treatment at the Zhejiang Provincial People’s Hospital on June 1, 2012, after a 6-day history of fever (maximum axillary temperature 40°C), malaise, chills, gingival bleeding, hyperemia of conjunctivae, and diarrhea (10 or fewer times per day). Initial laboratory testing revealed thrombocytopenia (13 × 10^9^ platelets/L; reference range 100–300 × 10^9^ platelets/L and leukocytopenia (0.93 × 10^9^ leukocytes/L; reference range 4–10 × 10^9^/L). Supportive therapy was provided, and the patient's condition seemed to improve on the second day: platelet count rose to 34 × 10^9^ platelets/L, and leukocyte count rose to 7.28 × 10^9^ leukocytes/L). However, on the third day, the patient became weak and died of multiple organ failure.

Serum samples from the patient were tested for the presence of SFTSV RNA by quantitative real-time reverse transcription PCR as previously described (*1*). The QIAamp Viral RNA Mini Kit (QIAGEN, Hilden, Germany) was used for RNA extraction. Detection of all 3 viral RNA segments by quantitative real-time reverse transcription PCR and isolation of the virus from Vero cell culture confirmed the association between the clinical syndrome and SFTSV infection. In addition, SFTSV-specific IgG and low levels of viral RNA were detected in a blood sample from a family member of the patient. The family member did not report exposure to potential animal hosts or vectors, so SFTSV transmission is believed to have occurred through personal contact when the family member was caring for the patient. Signs of illness did not develop in the family member.

**Figure 2:**
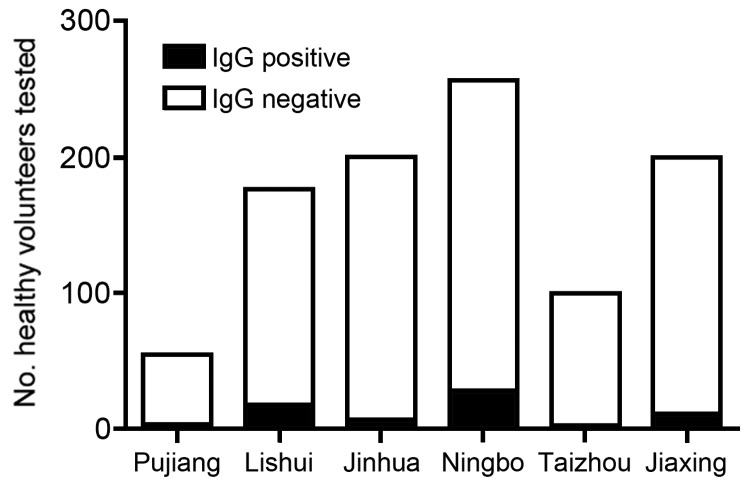
Seroprevalence of IgG to severe fever with thrombocytopenia syndrome virus in healthy persons from selected districts in Zhejiang Province, China, 2013.

To investigate if additional mild subclinical infections occurred, we, with the support of the local disease control department, conducted a seroprevalence study in the patient’s village in Pujiang district. A total of 54 blood samples were collected from 54 healthy volunteers. We used an ELISA kit provided by the National Center for Disease Control and Prevention to prepare and test serum samples for the presence of SFTSV-specific IgM and IgG (*5*,*6*). This ELISA compares well with serum neutralization assays for SFTSV (*6*). All serum samples were negative for SFTSV-specific IgM, whereas 4 (7.4%) of the serum samples were positive for SFTSV-specific IgG (Figure 2). None of the IgG-positive participants reported any disease symptoms that are associated with SFTSV infections.

To further investigate the occurrence of mild or subclinical SFTSV infections, we collected serum samples from healthy volunteers in 5 additional districts in Zhejiang Province and tested the samples for SFTSV-specific IgG. The percentages of positive samples, by district, follow (the no. positive/total no. tested are shown in parentheses): Lishui, 10.2% (18/176); Jinhua, 3.5% (7/200); Ningbo, 10.9 (28/256); Taizhou, 3% (3/100); and Jiaxing, 5.5% (11/200) (Figure 2). Results were confirmed by immunofluorescence assay conducted as previously reported (*1*). In brief, SFTSV-infected Vero cells were fixed with cold acetone, washed with distilled water, air-dried, and then stored at −70°C. Serum samples were diluted 1:20 in phosphate-buffered saline supplemented with 0.01% Evans blue (Sigma-Aldrich Co. LLC, St. Louis, MO, USA). Slides were then incubated with the diluted serum samples in a humidified chamber at 37°C for 30 min and then washed with phosphate-buffered saline. The wells were incubated with fluorescein isothiocyanate–labeled goat anti–human IgG conjugate (Boshide, Wuhan, China), washed, and analyzed by the use of fluorescence microscopy. The results of these experiments confirmed the presence of SFTSV-specific IgG in 84.5% (60/71) of the samples with ELISA-positive results. Serum samples that were negative for SFTSV by ELISA were also negative by immunofluorescence assay.

## Conclusions

SFTSV can cause severe disease and high rates of death among infected hospitalized patients. The virus also has the limited ability to be transmited from person to person through contact with contaminated blood, but secondary cases are generally less severe and have so far not resulted in fatalities (*7*–*9*). Nonetheless, there is great public health concern regarding SFTSV.

Our seroprevalence study was prompted by the identification of a subclinical, secondary infection that was most likely caused by person-to-person transmission of the virus from an infected family member with a fatal case of SFTS. We found an overall SFTSV seroprevalence of 7.2% among 986 healthy persons who reported no symptoms associated with SFTS. Because the seropositive participants in our study did not have contact with persons with diagnosed cases of SFTS, their infections most likely occurred through natural exposure. From this, we conclude that SFTSV infections are widespread in rural areas of Zhejiang Province, and only a small percentage of the infections result in clinical disease.
